# Inhibition of H1N1 by *Picochlorum* sp. 122 via AKT and p53 signaling pathways

**DOI:** 10.1002/fsn3.3110

**Published:** 2022-10-21

**Authors:** Danyang Chen, Zhihui Ning, Jingyao Su, Ruilin Zheng, Xia Liu, Hua‐lian Wu, Bing Zhu, Yinghua Li

**Affiliations:** ^1^ Center Laboratory, Guangzhou Women and Children's Medical Center Guangzhou Medical University Guangzhou China; ^2^ South China Sea Institute of Oceanology Chinese Academy of Sciences Guangzhou China

**Keywords:** apoptosis, influenza virus, Polysaccharides, signaling pathway

## Abstract

Influenza viruses cause a severe threat to global health, which can lead to annual epidemics and cause pandemics occasionally. However, the number of anti‐influenza therapeutic agents is very limited. Polysaccharides, extracted from *Picochlorum* sp. (PPE), seaweed Polysaccharides, have exhibited antiviral activity and were expected to be used for influenza treatment. In our research, the capability of PPE to inhibit H1N1 infection was proved in MDCK cells. PPE could make MDCK cells avoid being infected with H1N1 and inhibited nuclear fragmentation and condensation of chromatin. PPE evidently inhibited the generation of reactive oxygen species in MDCK cells. Mechanism study revealed that PPE prevented MDCK cells from H1N1 infection through induction of apoptosis by stimulating AKT signaling pathway and suppressing p‐p53 signaling pathway. In conclusion, PPE turns out to act as a prospective antiviral drug for H1N1 influenza.

## Graphical abstract

Influenza viruses cause a sever threat to global health, which can lead to annual epidemics and cause pandemics occasionally. Mechanism study revealed that PPE prevented MDCK cells from H1N1 infection through induction of apoptosis by stimulating AKT signaling pathway and supppressing p‐p53 signaling pathway. In conclusion, PPE turns out to act as an prospective antiviral drug for H1N1 influenza.

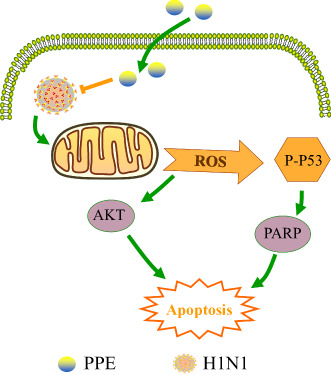

## 1 INTRODUCTION

Influenza A (H1N1), a new strain of swine origin, caused human infection in March 2009 and spread to a large number of different regions in a short period of time, becoming the most predominant circulating H1N1 influenza virus in humans today (Osztovits et al., 2009). H1N1 influenza virus, a kind of influenza A type virus, can cause highly infectious respiratory diseases, which was classified by two surface proteins: hemagglutinin (HA) and neuraminidase (NA; Li et al., 2018). NA inhibitors and M2 ion channel inhibitors approved by the US FDA are existing antiviral drugs (Chen & Guo, 2016; Lin et al., 2017). However, these antiviral drugs are effective only during early infection on patients (De Clercq, 2006; Nistal‐Villán & García‐Sastre, 2009). In addition, the occurrence of drug resistance, rimantadine, and AM are not used as common clinical drugs against influenza anymore. Thus, prompter of new antiviral drugs is needed in an attempt to control these influenza, while the availability of influenza vaccines shows little effect against the infection (Feng et al., 2014; Hu et al., 2022; Shang et al., 2021). There are many important compounds that can be synthesized by Algal products, polysaccharide (algae‐based polymer), lipid, and organic pigment, for example. These have attracted kinds of attention in industrial applications (Schutter et al., 2010). Besides, Algal is used as food supplements, aquaculture ingredients, animal feed, and soil biofertilizers (Li et al., 2013; Lian et al., 2018; Nie et al., 2016). Seaweed polysaccharide can be served as many important materials used in clinical practice, including anti‐inflammatory activity, anticancer activity, wound dressings, drug delivery, tissue engineering, immunomodulators, antibacterial effects, and anticoagulant activity, which are attributed to their rich sources, high safety, great biological activity, and low side effects (de Jesus Raposo et al., 2015; Kholiya et al., 2020; Okimura et al., 2020). Polysaccharides have shown the capability to inhibit viral infection in some studies, and they mainly inhibit virus from attaching cell wall to make cells avoid being invaded by virus and inhibit virus proliferation (Abu‐Galiyun et al., 2019; Liang et al., 2021; Sharma et al., 2021). The source of PPT as previously described (Guo et al., 2021; Xie et al., 2017; Xu et al., 2021; Yao et al., 2022). However, there are few reports about the antiviral activity. The mechanism of antiviral drugs against H1N1 infection has attracted researchers' attention increasingly. Li reported that functionalized selenium nanoparticles decorated with amantadine were reported to inhibit H1N1‐induced apoptosis via the ROS‐mediated AKT signaling pathway (Li et al., 2018). Wang found that Selenium Nanoparticles decorated with β‐Thujaplicin could inhibit apoptosis induced by H1N1 influenza virus via ROS‐mediated p53 and AKT Signaling Pathways (Wang et al., 2020). Mou discovered that EGCG induces β‐defensin 3 against H1N1 by the MAPK signaling pathway (Ha et al., 2022; Mou et al., 2020; Zhu et al., 2022). The anti‐H1N1 function of PPE was demonstrated, and the apoptosis mechanism referring to ROS‐mediated signaling pathways was discovered in the research. Our research showed that PPE inhibited host cell apoptosis induced by H1N1 through the AKT, P53, and PARP signaling pathways.

## 2 MATERIALS AND METHODS

### 2.1 Materials

PPE was acquired from the Chinese academy of South China Sea Institute of Oceanology, Chinese academy of sciences. MTT, Annexin V‐FLUOS, PI, TUNEL, and DAPI staining kits were obtained from Beyotime Biotechnology. All kits and antibodies used in Western blot analysis were purchased from Cell Signaling Technology (CST).

### 2.2 Cell and virus proliferation

MDCK cells were acquired from ATCC (CCL‐34TM) and cultured in Dulbecco's modified Eagle's medium (DMEM) containing 10% fetal bovine serum, 1% penicillin as well as 1% streptomycin at 37°C. H1N1 influenza virus was provided by the Virus Laboratory, Guangzhou Institute of Pediatrics, Guangzhou Women and Children's Medical Center, Guangzhou Medical University. H1N1 infection and 50% tissue culture infective dose (TCID_50_) were evaluated by Reed and Muench assessment, as shown in Table 1 (Molloy et al., 2013).

**TABLE 1:** Titer of H1N1

Dilution of H1N1	CPE	None CPE	Accumulation		The ratio of CPE (%)
			CPE	None CPE	
10^−1^	8	0	43	0	100 (43/43)
10^−2^	8	0	35	0	100 (35/35)
10^−3^	8	0	27	0	100 (27/27)
10^−4^	8	0	19	0	100 (19/19)
10^−5^	5	3	11	3	79 (11/14)
10^−6^	4	4	6	7	46 (6/13)
10^−7^	1	7	2	14	13 (2/16)
10^−8^	1	7	1	21	5 (1/22)
10^−9^	0	8	0	29	0 (0/29)
10^−10^	0	8	0	37	0 (0/37)
10^−11^	0	8	0	45	0 (0/45)
10^−12^	0	8	0	53	0 (0/53)

*Note*: The Reed‐Muench assay was used to test the titer of H1N1 (TCID50 = 10^−5.88^).

### 2.3 Cell viability

MTT assay was used to test the cytotoxicity of PPE as previously described (Lin et al., 2020). MDCK cells were inoculated at 5 × 10^4^/well and then cultured for 24 h. After that, H1N1 viruses were added with titer of 100 TCID_50_ for 2 h and removed which out of the cells. The cells were treated with H1N1 for 24 h before rinsed twice with PBS, and each well were added with 15 μl of MTT (5 mg/ml) solution. After 4 h, the formed formazan crystals were dissolved by dimethyl sulfoxide (DMSO) at 150 μl/well and detected at 570 nm.

### 2.4 Annexin‐V/PI double staining assay

MDCK cells were cultured and harvested as described previously (Xia et al., 2017). Briefly, the collected cells were suspended and then centrifuged. The supernatant solution was removed and the cells were suspended again. Finally, the cells were stained with the kits for 10–20 min without light and tested by flow cytometry, and Cell Quest VTM software was used to analyze the data.

### 2.5 The assessment of reactive oxygen species (ROS)

ROS in MDCK cells were detected as previously described (Lin et al., 2018). In brief, after H1N1‐infected MDCK cells were exposed to PPE for 24 h, MDCK cells were stained by the kits, and the results were tested with fluorescence microscope and fluorescence plate reader.

### 2.6 Test of mitochondrial membrane potential (△Ψm)

JC‐1 monomers was used to detect mitochondrial membrane potential (△Ψm) (Lin et al., 2020). H1N1‐infected MDCK cells were stained with JC‐1 without light for 20 min after exposed to PPE for 24 h. Flow cytometry was utilized.

### 2.7 TUNEL and DAPI staining

Effects of PPE on DNA fragmentation induced by H1N1 were detected by the kits following the protocol. Briefly, MDCK cells were labeled by TUNEL and nuclear was stained by DAPI. Then, the stained cells were observed with a fluorescence microscope (Nikon Eclipse 80i).

### 2.8 Determination of cytokine profiles

All kinds of cytokines shown were determined by commercial ELISA sets (BD OptEIATM, Becton Dickinson) following the manufacture protocol.

### 2.9 Statistical analysis

All the data are presented as mean ± SD. Differences between the two groups were evaluated using two‐tailed Student's *t*‐test. One‐way analysis of variance was used in multiple‐group comparisons. Differences were considered statistically significant with *p* < .05 (*) or *p* < .01 (**).

## 3 RESULTS AND DISCUSSION

### 3.1 In vitro antiviral effect of PPE

**FIGURE 1:**
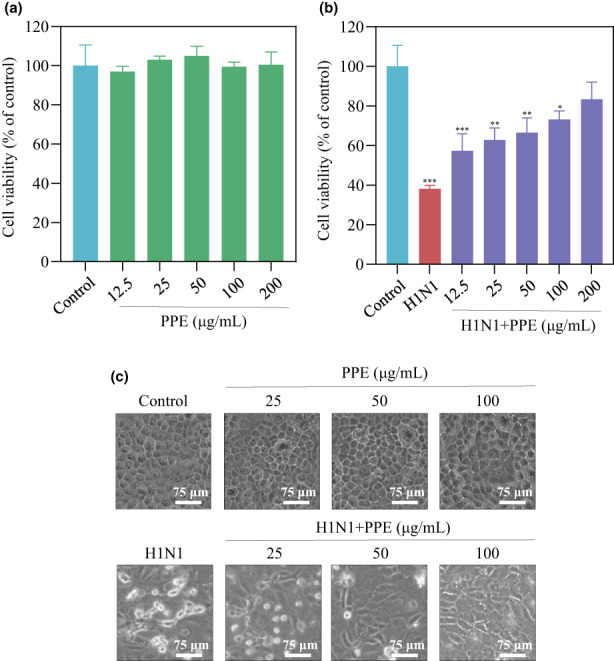
(A) The cytotoxicity of PPE against MDCK cells. (B and C) Effects of PPE on the growth of H1N1‐infected MDCK cells.

Cell viability was tested to assess PPE on antiviral effects by MTT assay. In Figure 1a, PPE exhibited low cytotoxicity against MDCK cells. In Figure 1b, MDCK was treated with PPE at concentrations of 12.5, 25, 50, 100, and 200 μg/ml after H1N1 infection, showed cell viabilities of 57.4%, 62.8%, 66.5%, 73.25%, and 83.4%, respectively. Thus, the protective effects on cell proliferation depend on PPE dose. As shown in Figure 1c, H1N1‐infected MDCK cells showed the cytopathic effects (CPEs), including cell numbers decrease, cell‐to‐cell contact reduction, and cytoplasmic shrinkage. However, the CPEs were weakened by PPE. In conclusion, PPE significantly protected MDCK cells from being infected by H1N1.

### 3.2 Effects of PPE on mitochondrial function of H1N1‐infected MDCK cells

**FIGURE 2:**
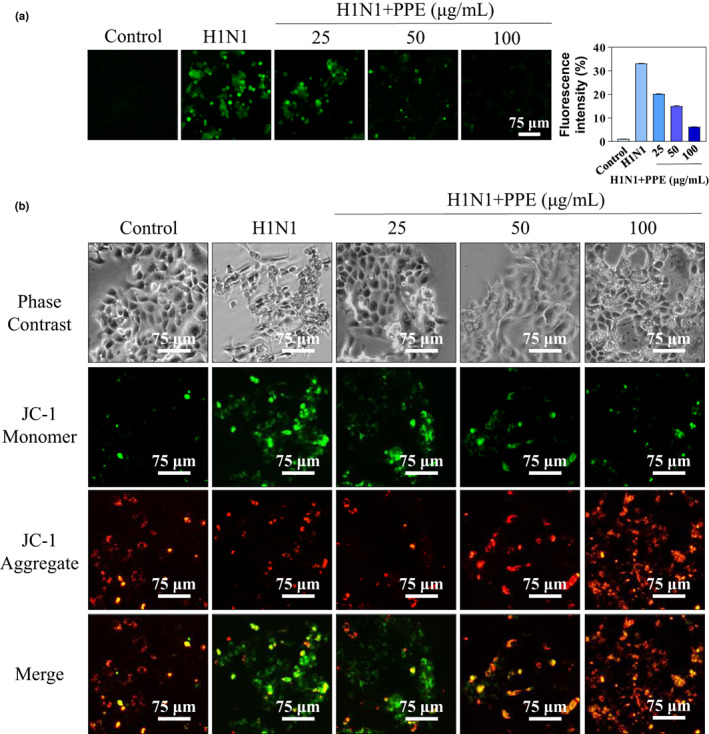
(A) Effects of PPE on ROS production of H1N1‐infected MDCK cells. (B) Effects of PPE on the mitochondria depolarization of H1N1‐infected MDCK cells.

Reactive oxygen species (ROS) are proved as a significant regulator of cell apoptosis induced by chemotherapy drugs. Mitochondrion acts as an important organelle to produce intracellular ROS. Overproduction of ROS can reduce ATP synthesized by mitochondria and result in mitochondrial dysfunction which leads to cell apoptosis further (Li et al., 2011). As shown in Figure 2a, H1N1 infection resulted in a significant elevation of green fluorescence of MDCK cells in both figures, which indicated that H1N1 induced mitochondrial dysfunction through ROS overproduction. As shown in Figure 2b, the green fluorescence of MDCK cells infected with H1N1 was enhanced and the mitochondrial membrane potential was decreased. After PPE treatment, the mitochondrial membrane potential of MDCK cells infected with H1N1 increased gradually. These results indicated that PPE might suppress the apoptotic pathway in MDCK cells via strengthening mitochondrial function.

### 3.3 Effects of PPE on apoptosis in H1N1‐infected MDCK cells

**FIGURE 3:**
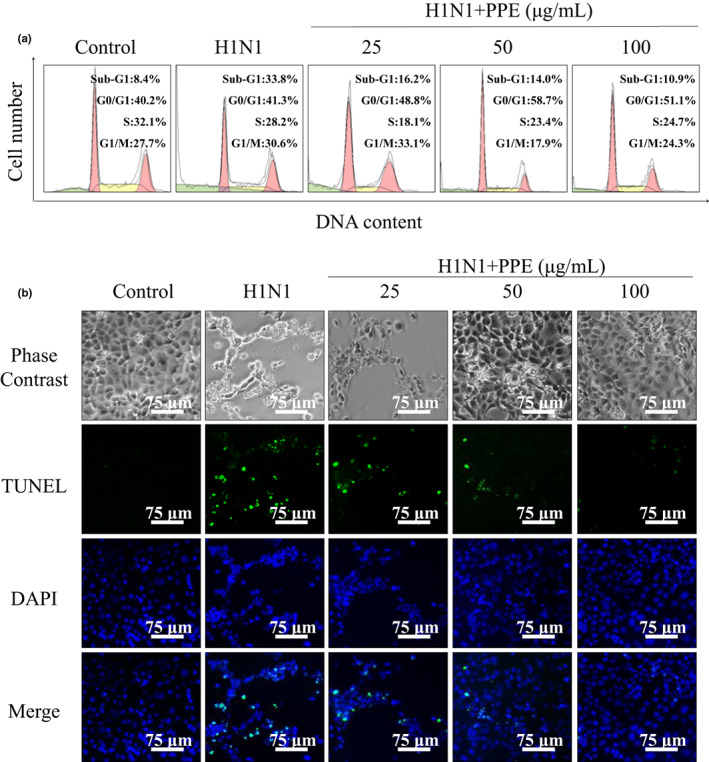
Effects of PPE on DNA fragmentation induced by H1N1 in MDCK cells.

In order to explore whether apoptosis was induced in H1N1‐infected MDCK cells and effects of PPE, further steps were taken. As shown in Figure 3a, there is an obvious sub‐G1 peak (33.8%), compared to the control group (8.4%). The sub‐G1 peak, representing apoptosis of cells, was lower in the concentration of 100 μg/ml group than other groups with lower concentrations, suggesting that PPE with higher concentrations showed a greater capability to inhibit apoptosis. Significant differences were not observed in cell cycle distribution. DNA fragmentation is one of the representatives of cell apoptosis (Moosavi et al., 2018). The apoptosis induced by H1N1 was further confirmed by TUNEL and DAPI assay. As shown in Figure 3b, H1N1‐infected MDCK cells showed typical apoptotic features, such as nuclear condensation and DNA fragmentation. However, the cells showed less nuclear condensation and DNA fragmentation features after treatment with PPE. The results demonstrated that PPE could inhibit apoptosis of H1N1‐infected MDCK cells.

### 3.4 Effects of PPE on the early and late apoptosis of H1N1‐infected MDCK cells

**FIGURE 4:**
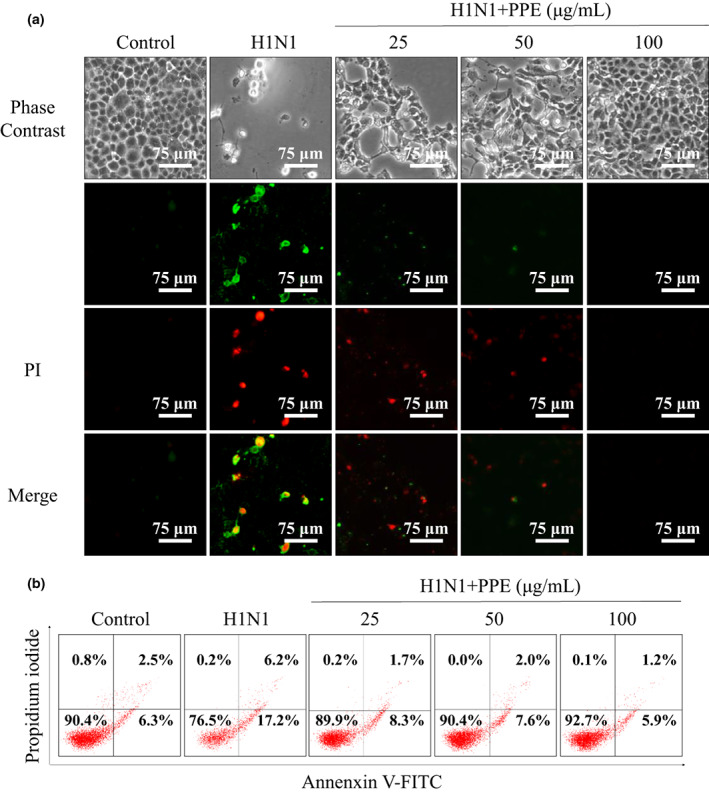
Effects of PPE on the early and late apoptosis of H1N1‐infected MDCK cells.

Annexin‐V/PI double staining acts as a more effective way for cell apoptosis examination (Yu et al., 2020). As shown in Figure 4a, H1N1 infection resulted in a significant elevation of green and red fluorescence of MDCK cells, which indicated that H1N1 induced early and late apoptosis of H1N1‐infected MDCK cells. After treatment with PPE, the double fluorescence showed a decrease obviously. As shown in Figure 4b, the group with 100 μg/ml PPE significantly inhibited MDCK cells apoptosis, and led to lower cell apoptosis proportion (early apoptosis: 5.9% and late apoptosis: 1.2%), compared to H1N1‐infected group (17.2% and 6.2%), group with 25 μg/ml PPE (8.3% and 1.7%), or group with 50 μg/ml PPE (7.9% and 2.0%). The results indicated that PPE suppressed viral activity in a dose‐dependent manner, and exhibited great antiviral capability to prevent MDCK cells from undergoing apoptosis in vitro.

### 3.5 The innate immune response modulation of PPE to H1N1 infection

**FIGURE 5:**
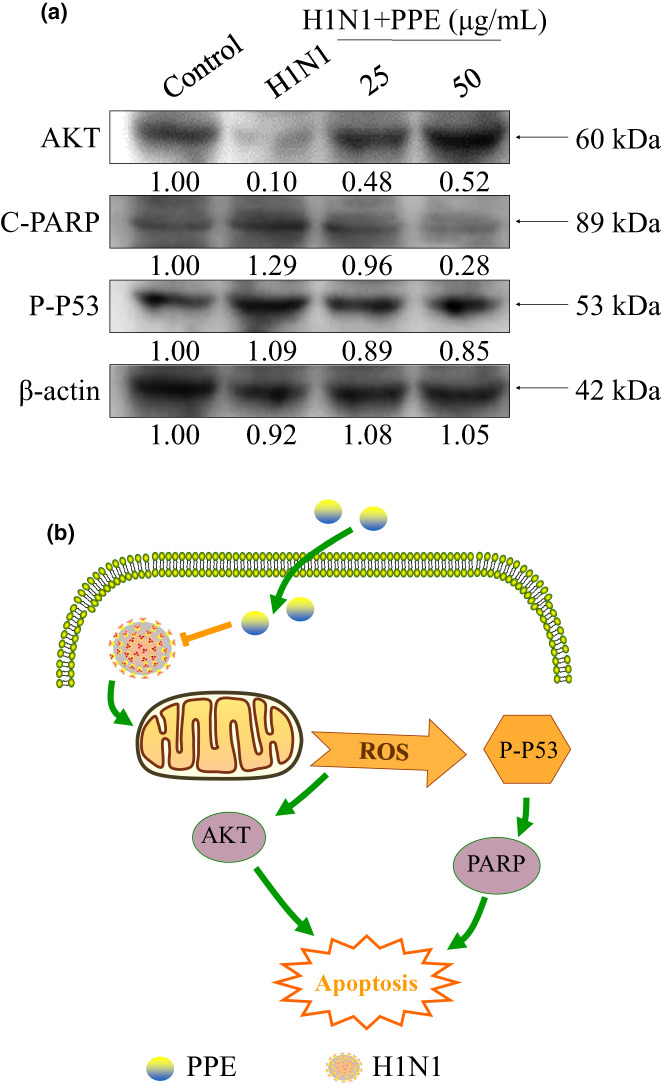
Intracellular apoptotic signaling pathways by PPE in H1N1 infection of MDCK cells.

In addition, PPE exerted an anti‐inflammatory activity. In Figure 5, MDCK cells showed a basal secretion of cytokines. H1N1 infection induced a strong innate immune response in the hosts such as TNF‐α, IL‐2, IL‐17F, IL‐4, IL‐17A, and IL‐1β, while hosts treated with PPE showed significant suppression of innate immune response.

### 3.6 Effects of PPE on apoptotic signaling pathways activated by ROS

**FIGURE 6:**
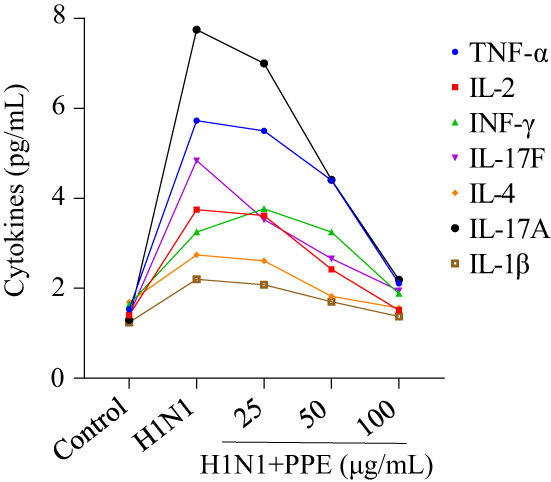
Effects of PPE on inflammatory response of H1N1‐infected MDCK cells.

The overexpression of ROS could cause damage to DNA via regulating apoptosis signaling pathways. In Figure 6a, compared to H1N1‐infected MDCK cells, infected cells treated with PPE obviously decreased the expression quantity of Cleaved‐PARP. As a transcription factor, P53 can activate the expression of various genes. The target genes of P53 were directly involved in the regulation of cell cycle, repair of DNA damage, and other life processes. PARP was involved in many important life activities such as apoptosis, transcription, and DNA repair, and its activation was regulated by apoptotic gene P53. H1N1 infection led to intracellular oxidative damage and phosphorylation of P53, which then mediated PARP cleavage and increased the expression of Cleaved‐PARP. At the same time, the quantity of Akt was significantly increased by PPE and the quantity of p‐p53 was significantly decreased subsequently. AKT played an important role in mitochondria‐mediated apoptosis pathway and can regulate cell apoptosis through phosphorylation or interaction with cytokines. In conclusion, the results indicated that PPE suppressed cell apoptosis induced by H1N1 via ROS‐mediated cleaved‐PARP, AKT, and P‐P53 signaling pathways (Figure 6b).

## 4 CONCLUSIONS

In our research, the antiviral activity and mechanisms of PPE were explored by different methods. The research demonstrated that PPE obviously restrained H1N1 proliferation and reduced apoptosis of MDCK cells. The mechanisms revealed that PPE limited H1N1‐induced apoptosis of hosts through AKT and p53 signaling pathways, and suppressed the innate immune response of MDCK cells induced by H1N1. Our findings revealed that PPE could be a prospective drug to treat H1N1 infection.

## CONFLICT OF INTEREST

The authors report no conflicts of interest in this work.

## Data Availability

Data sharing is not applicable to this article as no new data were created or analyzed in this study.
